# Body Dissatisfaction and Binge Eating: The Moderating Roles of Sweet Taste Reward Sensitivity and Dietary Restraint among Tobacco Product Users

**DOI:** 10.3390/ijerph192315523

**Published:** 2022-11-23

**Authors:** Tyler B. Mason, Anna Dolgon-Krutolow, Kathryn E. Smith, Adam M. Leventhal

**Affiliations:** 1 Department of Population and Public Health Sciences, University of Southern California Keck School of Medicine, Los Angeles, CA 90032, USA; 2 Institute for Addiction Science, University of Southern California, Los Angeles, CA 90032, USA; 3 Department of Psychiatry and Behavioral Sciences, University of Southern California, Los Angeles, CA 90032, USA

**Keywords:** binge eating, body dissatisfaction, restraint, sweet taste, moderation

## Abstract

Body dissatisfaction is a key predictor of binge eating, yet less is known about factors that may potentiate the association between body dissatisfaction and binge eating. This study examined self-reported dietary restraint and sweet taste reward sensitivity as candidate moderators of the association between body dissatisfaction and binge eating in adults. A convenience sample of 221 tobacco product users completed measures of eating disorder pathology and sweet taste reward sensitivity. Results revealed that elevated sweet taste reward sensitivity strengthened the positive association between higher body dissatisfaction and binge eating. However, there was no main effect, or moderation effect, of dietary restraint on binge eating. The findings of this study demonstrate the key role of sweet taste reward sensitivity in potentiating the association between body dissatisfaction and binge eating. Sweet taste reward sensitivity may serve as a key dispositional factor for uncontrolled eating.

## 1. Introduction

Binge eating symptoms occur across eating disorders and involve eating large amounts of food in a short period of time and feelings of a subjective sense of loss of control and overeating [1]. Of critical importance, binge-eating symptoms are associated with poor psychosocial and physical health outcomes, notably psychiatric disorders, obesity, and poor quality of life and social functioning [2,3,4]. While binge-eating symptoms may be elevated among those with obesity, binge-eating symptoms affect individuals of all body sizes and weights [5].

Body dissatisfaction is a central risk and maintenance factor included in most theoretical models of binge eating [6], and empirical research has found positive cross-sectional and prospective associations between elevated body dissatisfaction and binge eating [7,8,9]. In fact, regardless of weight status, body image concerns were a key factor that distinguished between women with and without a binge-eating disorder [10,11]. Yet, body dissatisfaction is relatively common in today’s society, often termed “normative discontent” [12]. As such, more research is needed to understand factors that may potentiate, or moderate, the association between body dissatisfaction and binge eating to better understand the dynamics of the association between body dissatisfaction and binge eating. Two key candidate moderators that have been argued to be important drivers of binge eating in the eating disorders and food addiction literature are dietary restraint and sweet taste reward sensitivity [13,14].

The restraint model of binge eating suggests that individuals who exhibit elevated dietary restraint, or attempts to control or limit their dietary intake for the purpose of weight control, are more likely to engage in binge eating [15]. Some empirical tests of the restraint model have shown positive associations between dietary restraint and binge eating [16]. However, Spoor and colleagues [17] did not find a prospective association between dietary restraint and later binge eating, and other research found that in a multivariable model with restraint and body dissatisfaction, only body dissatisfaction was significantly associated with bulimic symptoms [18]. Nevertheless, several studies indicate that dietary restraint moderates the association between psychosocial factors and binge eating [19,20,21]. For example, Woods and colleagues [21] found that dietary restraint strengthened the positive association of daily and life stressors on binge eating symptoms. These studies support the possibility of dietary restraint as a moderator of the association between body dissatisfaction and binge eating, such that individuals with greater body dissatisfaction who engage in dietary restraint as a means of altering their shape/weight may be at greatest risk for subsequent binge eating.

In addition to restraint, reward-related processes have been implicated in binge eating, including food liking, wanting, and expectancies [22,23,24]. For some people, sweet-tasting food may be particularly rewarding [25]. In addition, animal studies have found responsiveness to sweet taste to be particularly important to binge eating in rats [13,26]. While less research has studied sweet taste reward sensitivity in relation to binge eating in humans, there have been several studies examining sweet taste reward-related measures in individuals with binge-eating spectrum disorders [27,28]. One recent study of adults with binge-eating disorder found that elevated sweet taste preference was associated with higher binge-eating and overeating frequencies [28]. Another study showed that women with bulimia nervosa reported elevated sweet taste pleasantness compared to healthy controls [27]. Furthermore, food addiction and food responsiveness measures have shown positive associations with increased binge eating [29,30,31]. This available research suggests that sweet taste reward sensitivity may play a role in binge eating, but the moderating role of sweet taste reward sensitivity in the association between psychological factors, including body dissatisfaction, and binge eating has not been examined. Similar to restraint, it is possible that individuals with greater body dissatisfaction who report higher sweet taste reward sensitivity are at the greatest risk for subsequent binge eating.

To better understand the psychological processes that potentiate the association between body dissatisfaction and binge eating, a secondary analysis of an existing dataset was conducted. Research questions in the current study were developed post hoc to the original aims of the parent study. The current study examined the moderating roles of self-reported dietary restraint and sweet taste reward sensitivity in the association between body dissatisfaction and binge eating. It was hypothesized that elevated sweet taste reward sensitivity and dietary restraint would separately strengthen the positive association between body dissatisfaction and binge eating.

## 2. Method

### 2.1. Participants

The current paper used a convenience sample of United States (U.S.) adults who currently used tobacco products. Data were collected from 221 adults who either currently used e-cigarettes or only used combustible cigarettes but were interested in trying e-cigarettes. Participants were recruited through internet advertisements in the U.S. Procedures were reviewed and approved by the University of Southern California Institutional Review Board. Eligible and interested participants (assessed via phone screen) completed a virtual study visit, where they provided written informed consent. Participants completed baseline survey questionnaires and an experimental e-cigarette product appeal paradigm. The questionnaires were completed during breaks from the e-cigarette product testing. Only self-report questionnaires were used in the present analyses.

### 2.2. Measures

Eating Pathology Symptoms Inventory (EPSI) [32]. The EPSI was used to assess several facets of eating disorder psychopathology. The current study used Binge Eating (e.g., “I stuffed myself with food to the point of feeling sick”), Body Dissatisfaction (e.g., “I wished the shape of my body was different”), and Dietary Restraint (e.g., “I tried to exclude “unhealthy” foods from my diet”) subscales. Participants were asked to report how frequently they experienced each item during the past four weeks on a 5-point scale, from 0 (*never*) to 4 (*very often*). Scores were generated by averaging the responses across items for each of the three subscales. The EPSI has shown excellent psychometric properties in previous research across gender [32,33]. The Cronbach’s alphas in the current study were 0.89, 0.73, and 0.83 for body dissatisfaction, dietary restraint, and binge eating, respectively.

Sweet Taste Questionnaire (STQ) [34]. The STQ is a 12-item self-report measure that assesses sweet taste reward sensitivity with items assessing behaviors and attitudes. Items from the STQ measure sensitivity to the mood-altering effects of sugary foods, as well as impaired control over the consumption of sweet foods. Sample items include, “I often have an urge for something sweet”, “I have problems controlling how much sweet food I eat”, and “I am less irritable if I have something sweet to eat.” Participants responded to each item using a 7-point scale ranging from 1 (*strongly disagree*) to 7 (*strongly agree*). Kampov-Polevoy et al. [34] reported good psychometric properties of the STQ. The Cronbach’s alpha in the current study was 0.88.

### 2.3. Statistical Analyses

Analyses were run in SPSS v 28.0 (IBM; Armonk, NY, USA). Descriptive statistics and bivariate correlations were calculated for the study variables. Next, Q-Q plots, skewness, and kurtosis statistics were examined to test for normality [35]. Linear multiple regression was run within the SPSS PROCESS Macro [36], which is appropriate for examining interactions (i.e., moderation) between continuous independent variables. A two independent moderators model was conducted, which included examining the main effects of body dissatisfaction, dietary restraint, and sweet taste reward sensitivity and two-way interactions between body dissatisfaction and dietary restraint and body dissatisfaction and sweet taste reward sensitivity in relation to binge eating. All independent variables used to create interaction terms were centered so that the mean was 0, which helps avoid issues with multicollinearity. Variance inflation factor (VIF) statistics were examined to test for multicollinearity, with VIF > 10 indicating potential multicollinearity [37]. Covariates included gender, age, and BMI. Significance testing was conducted using bootstrap confidence intervals (CIs). If the bootstrap CI did not include 0, then the estimate was significant. To probe significant interactions, interactions were plotted, and conditional effects were calculated.

## 3. Results

The gender makeup of the sample was 45.7% Female (*n* = 101), 53.3% Male (*n* = 118), and 0.9% Other (*n* = 2). The race/ethnicity of the sample was 67.1% White, 13.2% Black, 4.6% Asian or Pacific Islander, 0.9% American Indian or Alaskan Native, 5.5% Hispanic, 0.9% Other, and 7.8% Multiracial. Bivariate correlations and descriptive statistics are reported in Table 1. Age was significantly positively correlated with higher BMI and lower binge eating, and BMI was significantly positively correlated with greater body dissatisfaction. Body dissatisfaction was significantly positively correlated with greater dietary restraint, sweet taste reward sensitivity, and binge eating. Dietary restraint was not significantly correlated with sweet taste reward sensitivity or binge eating, but sweet taste reward sensitivity was significantly positively correlated with higher levels of binge eating. Q-Q plots and skewness and kurtosis statistics indicated normality for body dissatisfaction, dietary restraint, sweet taste reward sensitivity, and binge eating.

The linear multiple regression is reported in Table 2. VIF values were all below 1.5, indicating no issues with multicollinearity. Younger age and male compared to female gender were significantly associated with greater binge eating; BMI was unrelated to binge eating. With regard to the main effects, greater body dissatisfaction and sweet taste reward sensitivity were significantly associated with higher binge eating. There was no significant main effect of dietary restraint in relation to binge eating. There was a significant interaction between body dissatisfaction and sweet taste reward sensitivity in relation to binge eating, but no significant interaction between body dissatisfaction and dietary restraint with binge eating. The model explained 31% of the variance in binge eating (*R*^2^ = 0.31).

**Figure 1:**
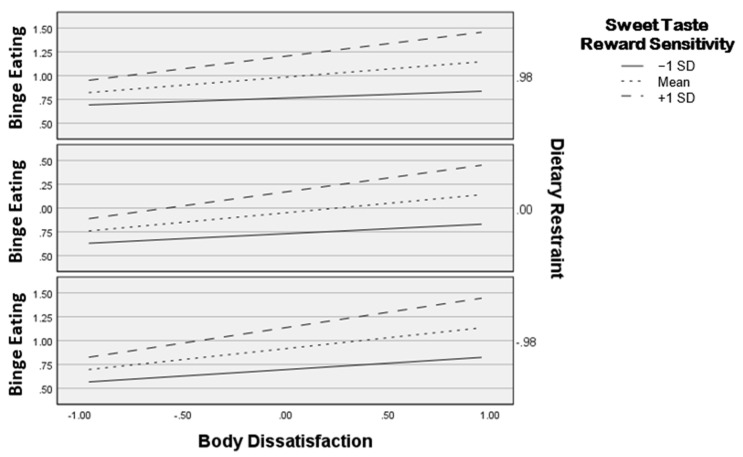
Interaction between body dissatisfaction and sweet taste reward sensitivity in relation to binge eating plotted at one standard deviation below the mean (−1 SD), mean, and one standard deviation above the mean (+1 SD) levels of sweet taste reward sensitivity. The three boxes indicate one deviation below the mean (−0.98), mean (0), and one standard deviation above the mean (0.98) levels of dietary restraint.

The interaction of body dissatisfaction and sweet taste reward sensitivity is displayed in Figure 1. As shown, higher sweet taste reward sensitivity strengthened the positive association between body dissatisfaction and binge eating. Table 3 displays the conditional effects of body dissatisfaction on binge eating at centered values of sweet taste reward sensitivity and dietary restraint. Regardless of levels of dietary restraint, conditional effects analyses revealed that there was a positive association between body dissatisfaction and binge eating at mean and +1 SD levels of sweet taste reward sensitivity. There was no significant association at −1 SD of sweet taste reward sensitivity.

## 4. Discussion

This study examined the moderating roles of dietary restraint and sweet taste reward sensitivity in the association between body dissatisfaction and binge eating. Consistent with theoretical and empirical work [6,7,8,9], higher body dissatisfaction was associated with greater levels of binge eating. Dietary restraint was not associated with binge eating and did not moderate the association between body dissatisfaction and binge eating; which is contrary to the restraint model of binge eating [15]. Yet, sweet taste reward sensitivity was positively associated with binge eating and strengthened the association between body dissatisfaction and binge eating. These results suggest that among a non-clinical sample of adult tobacco product users, over-responsiveness to sweet taste appears to be more important in relation to binge eating compared to dietary restraint.

Sweet taste reward sensitivity strengthened the association between body dissatisfaction and binge eating. As such, the experience of elevated body dissatisfaction and sweet taste reward sensitivity represents a psychological profile associated with the risk of more binge-eating symptoms. This supports the notion of the combined importance of eating-disorder-related risk factors (e.g., body dissatisfaction) and food addiction-related risk factors (e.g., responsiveness to sweet taste) in predicting risk for elevated binge-eating symptoms [14]. Studies show that binge-eating episodes often involve sweet foods, and thus, individuals with elevated sweet taste reward sensitivity may lose control when eating sweet types of food [38,39,40]. Moreover, individuals with elevated body dissatisfaction may experience maladaptive intrusive food-related thoughts and preoccupations [7,41,42]. Given the results of the current study, intrusive food-related thoughts may be exacerbated by elevated reward sensitivity to sweet food, which could be associated with a greater risk of binge eating.

The lack of a moderating role of dietary restraint is inconsistent with prior research, as several studies have shown dietary restraint to be a moderator of non-specific eating disorder risk factors and binge eating, such as daily stress and negative affect [19,20,21]. Given the relationship between body dissatisfaction and dietary restraint, these findings could be explained by body dissatisfaction being a confounding variable. That is, body dissatisfaction may be the key moderator associated with binge eating, rather than dietary restraint, which is supported by a previous study [9]. Furthermore, the association between restraint and binge eating appears to be quite complex, with studies finding nuanced associations between restraint and binge eating when examining within- and between-subject associations [43,44]. Additionally, there are a host of conceptualizations and measures of restraint, which include both adaptive and maladaptive components. As such, various conceptualizations and measures of dietary restraint might have differential relationships with binge eating [45]. It is also possible that this finding is related to the sample composition of tobacco product users. Given the overlap between substance use and food addiction, tobacco product users may be more likely to be influenced by addiction-related mechanisms, such as reward sensitivity, rather than eating disorder-specific mechanisms, such as dietary restraint [46].

Several limitations must be noted. First, this was a cross-sectional study, so causality and directionality cannot be confirmed. Longitudinal research will be needed to understand causal relationships between these variables. Second, this study used a convenience sample of tobacco product users. Thus, further research is necessary to determine whether the current findings generalize to other samples, as well as the differences between tobacco vs. non-tobacco product users. Yet, the current findings are important given the associations between tobacco product use and eating disorder psychopathology, and related behaviors, such as binge eating [47]. Third, all measures were assessed with retrospective self-report questionnaires, which are subject to reporting biases. Future research should utilize other methodologies that limit self-report biases, such as intensive longitudinal and experimental designs. Specifically, assessing responses to actual sweet taste intake is an important measure of sweet taste reward sensitivity to include in future research. Fourth, this study focused on sweet taste reward sensitivity, yet binge-eating episodes may consist of other types of foods, such as savory or high-fat foods, and more research will be needed to understand relationships between responsiveness to other types of foods and binge eating.

In conclusion, individuals with elevated body dissatisfaction reported higher binge-eating symptoms when they also had elevated sweet taste reward sensitivity. Models of binge eating should integrate constructs from the eating disorder and food addiction literature to best understand the risk of engaging in binge eating. In addition, dietary restraint was not related to binge-eating symptoms, and future studies of integrative models of binge eating should clarify the role of restraint in these models.

## Figures and Tables

**Table 1:** Pearson Correlations and Descriptive Statistics of Study Variables.

	1	2	3	4	5	6
1. Age	-	0.17 *	−0.04	−0.09	0.06	−0.22 ***
2. Body mass index		-	0.29 ***	0.13	0.09	0.001
3. Body dissatisfaction			-	0.21 **	0.34 ***	0.32 ***
4. Dietary restraint				-	0.06	0.12
5. Sweet taste reward sensitivity					-	0.40 ***
6. Binge eating						-
						
M	34.53	26.91	1.25	1.42	2.92	0.98
SD	13.28	6.11	0.96	0.98	1.39	0.70
Minimum	21	16.44	0.00	0.00	1.00	0.00
Maximum	73	52.37	3.86	4.00	6.92	3.71
Skewness	0.88	1.31	0.58	0.62	0.62	1.10
Kurtosis	−0.26	2.56	−0.44	−0.12	−0.27	1.44

Note. * *p* < 0.05, ** *p* < 0.01, *** *p* < 0.001.

**Table 2:** Linear Regression Model of Binge Eating on Body Dissatisfaction, Dietary Restraint, and Sweet Taste Reward Sensitivity.

Parameter	B	SE	*p*	95% CI
Intercept	1.30	0.22	<0.001	[0.88, 1.73]
Age	−0.01	0.003	0.006	[−0.02, −0.003]
BMI	−0.007	0.007	0.33	[−0.02, 0.007]
Male vs. female	0.27	0.09	0.004	[0.09, 0.45]
Other vs. female	−0.35	0.43	0.41	[−1.19, 0.49]
Body dissatisfaction	0.20	0.05	0.002	[0.10, 0.30]
Dietary restraint	0.04	0.04	0.41	[−0.05, 0.12]
Sweet taste reward sensitivity	0.16	0.03	<0.001	[0.10, 0.22]
Body dissatisfaction × dietary restraint	−0.04	0.04	0.47	[−0.11, 0.05]
Body dissatisfaction × sweet taste reward sensitivity	0.07	0.03	0.02	[0.01, 0.13]

**Table 3:** Conditional Effects of Body Dissatisfaction at Values of Sweet Taste Reward Sensitivity and Dietary Restraint.

Sweet Taste Reward Sensitivity	Dietary Restraint	Estimate	SE	*p*	95% CI
−1 SD	−1 SD	0.14	0.08	0.08	[−0.02, 0.29]
−1 SD	Mean	0.10	0.07	0.13	[−0.03, 0.24]
−1 SD	+1 SD	0.08	0.08	0.36	[−0.09, 0.24]
Mean	−1 SD	0.23	0.07	<0.001	[0.10, 0.36]
Mean	Mean	0.20	0.05	<0.001	[0.10, 0.30]
Mean	+1 SD	0.17	0.07	0.01	[0.04, 0.30]
+1 SD	−1 SD	0.32	0.08	<0.001	[0.17, 0.48]
+1 SD	Mean	0.29	0.06	<0.001	[0.17, 0.42]
+1 SD	+1 SD	0.26	0.07	<0.001	[0.12, 0.41]

Note. SD = standard deviation.

## Data Availability

The data are available from the authors by request.
